# The Role of *Pfmdr1* and *Pfcrt* in Changing Chloroquine, Amodiaquine, Mefloquine and Lumefantrine Susceptibility in Western-Kenya *P*. *falciparum* Samples during 2008–2011

**DOI:** 10.1371/journal.pone.0064299

**Published:** 2013-05-13

**Authors:** Fredrick L. Eyase, Hoseah M. Akala, Luiser Ingasia, Agnes Cheruiyot, Angela Omondi, Charles Okudo, Dennis Juma, Redemptah Yeda, Ben Andagalu, Elizabeth Wanja, Edwin Kamau, David Schnabel, Wallace Bulimo, Norman C. Waters, Douglas S. Walsh, Jacob D. Johnson

**Affiliations:** 1 Department of Emerging Infectious Diseases-Global Emerging Infections Surveillance and Response System (DEID-GEIS) Program, United States Army Medical Research Unit-Kenya (USAMRU-K), Nairobi, Kenya; 2 United States Military Academy, New York, New York, United States of America; 3 Armed Forces Research Institute of Medical Sciences, Bangkok, Thailand; 4 Walter Reed Army Institute of Research, Silver Spring, Maryland, United States of America; Weill Cornell Medical College, United States of America

## Abstract

Single Nucleotide Polymorphisms (SNPs) in the *Pfmdr1,* and *Pfcrt,* genes *of Plasmodium falciparum* may confer resistance to a number of anti-malaria drugs. *Pfmdr1* 86Y and haplotypes at *Pfcrt* 72-76 have been linked to chloroquine (CQ) as well as amodiaquine (AQ) resistance. mefloquine (MQ) and lumefantrine (LU) sensitivities are linked to *Pfmdr1* 86Y. Additionally, *Pfcrt* K76 allele carrying parasites have shown tolerance to LU. We investigated the association between *Pfmdr1* 86/*Pfcrt* 72-76 and *P. falciparum* resistance to CQ, AQ, MQ and LU using field samples collected during 2008–2011 from malaria endemic sites in western Kenya. Genomic DNA from these samples was genotyped to examine SNPs and haplotypes in *Pfmdr1* and *Pfcrt* respectively. Additionally, immediate *ex vivo* and *in vitro* drug sensitivity profiles were assessed using the malaria SYBR Green I fluorescence-based assay. We observed a rapid but steady percent increase in wild-type parasites with regard to both *Pfmdr1* and *Pfcrt* between 2008 and 2011 (p<0.0001). Equally, a significant reciprocate decrease in AQ and CQ median IC_50_ values occurred (p<0.0001) during the same period. Thus, the data in this study point to a significantly rapid change in parasite response to AQ and CQ in the study period. This may be due to releasing of drug pressure on the parasite from reduced use of AQ in the face of increased Artemisinin (ART) Combination Therapy (ACT) administration following the intervention of the Global Fund in 2008. LU has been shown to select for 76K genotypes, thus the observed increase in 76K genotypes coupled with significant cross resistance between LU and MQ, may herald emergence of tolerance against both drugs in future.

## Introduction

Until 1998, CQ was the drug of choice in Kenya due to its efficiency in tackling the malaria menace. However, resistance developed against CQ leading to replacement with the sulfadoxine/pyrimethamine (S/P) combination. Soon thereafter resistance also developed against SP [1] and was in turn replaced in 2006 with artemisinin (ART)-based derivatives as the primary malaria treatment medicine. Prior to the widespread adoption of ART based drugs, there was significant use of amodiaquine (AQ) as an over-the-counter medication because widespread resistance to S/P was observed clinically in Kenya [2]. Mono-therapies are highly susceptible to the development of resistance as exemplified by the recent emergence of resistance to artesunate monotherapy on the Thai-Cambodia border [3], [4]. Consequently antimalarial combination therapies have been adopted the world over in an effort to protect the available antiplasmodial drugs [5]. Currently in Kenya, ART combination therapy (ACT) is advocated for the treatment of uncomplicated malaria [6]. Some of the ACTs that have shown efficacy include artemether-lumefantrine (AL), which is the current first-line antimalarial in Kenya, artesunate-mefloquine [7] and artesunate-amodiaquine (ASAQ) [8]. Combination therapy is preferred because a short half life drug such as an ART derivative clears most of the parasites. Thus, even those parasites that may possess a level of resistance to the longer half life partner drug are killed. In reciprocity, the longer acting partner drug such as LU protects its partner by killing any residual parasites [9]. However, there is a potential time window–after the shorter half-life partner has been metabolized and only the longer life partner drug is circulating at low levels–when malaria reinfection may initiate the selection of drug resistance to the partner long-life drug.

The molecular mechanisms behind multidrug resistance by *P. falciparum* remain largely unknown. However, polymorphisms within the *Pfmdr1* gene that encodes a trans-membrane homologue of the PGH1 protein have been implicated. The main implicated *Pfmdr1* SNPs include N86Y, Y184F, S1034C, N1042D [10]. Some of the drugs affected by SNPs in *Pfmdr1* include AQ, LU, ART, MQ, and CQ. Resistance to AQ and its metabolite DEAQ has been linked to mutations in *Pfmdr1* [11], [12]. Whereas resistance to AQ has been extensively reported in South America [13], [14], this drug has remained relatively effective in Africa, especially as a viable partner drug for ART [15]. The selection by the AL combination for *Pfmdr1* alleles has recently been observed [16]. *Pfmdr1* N86 has also been associated with increased tolerance to the artemether and LU drugs separately [17], [18], [19]. Moreover, there are indications that *Pfmdr1* gene amplification may cause resistance to ART [20], [21]. Overall, amplification of the *Pfmdr1* gene leads to mefloquine resistance [22], [23]. Even though MQ has been adopted as partner drug to artemisinin, it has been observed to select for the wild type *Pfmdr1* N86 [24]. On the other hand, parasites with the mutant 86Y show increased sensitivity to MQ [25]. *Pfmdr1* involvement in CQ resistance has been suggested to be secondary to *Pfcrt* [26], [27]. Nevertheless, CQ selects for parasites with *Pfmdr1* 86Y mutation [26], thus showing an inverse relationship with MQ. Differences in CQ IC_50s_ of isolates with the same *Pfmdr1* and *Pfcrt* mutation profiles have been observed, indicating that there are other mechanisms besides those associated with the *Pfmdr1* and *Pfcrt* genes, involved in CQ resistance [28].

Mutations in *Pfcrt* are associated with CQ, AQ, and LU resistance; specifically, the *Pfcrt* 72-76 CVIET and SVMNT haplotypes have been implicated. The 76T point mutation is the main marker for CQ resistance, while the SVMNT haplotype is required for resistance against AQ. In the case of CQ, the mutated export protein loses a positive charge and, therefore, has ability to transport protonated CQ from the food vacuole [29]. In Malawi, a rapid decrease in parasites carrying 76T was observed after the official discontinuation of CQ use [30]. Parasites carrying the CVIET haplotype are moderately resistant to AQ and highly resistant to CQ. Inversely, parasites carrying the SVMNT haplotype are highly resistant to AQ, but only moderately resistant to CQ [31]. Additionally *Pfcrt* K76 has been linked to emerging LU tolerance [17], [19], [32].

Full implementation of the use of Coartem™, a fixed dose AL combination, as the official first-line antimalarial therapy in Kenya was achieved beginning 2008. Consequently, we examined *Pfmdr1* codon 86, *Pfcrt* codon 76, and the *Pfcrt* 72-76 haplotypes, in samples from Kisumu, Kisii and Kericho districts of western-Kenya, in relation to *in vitro* drug responses beginning 2008 until 2011. We hypothesized that, the current policy change had major implications on other drugs that had been in use until and during the time of the policy change. Therefore the relevance of the malaria genotypic and phenotypic sensitivity data for western Kenya as a result of the ACT policy implementation will be discussed.

## Materials and Methods

### Ethics Statement, Study Protocol, Sites and Subjects

The study protocol was approved by the Kenya Medical Research Institute (KEMRI, Protocol # 1300) and Walter Reed Army Institute of Research (WRAIR, Protocol # 1384) institutional review boards. Field isolates were obtained from Kenya Ministry of Health facilities, namely Kisumu, Kisii and Kericho district hospitals. We enrolled subjects attending outpatient clinics between 2008 and 2011, who were at least 6 months old and were suspected to have un-complicated *P. falciparum* malaria. Written informed consent was obtained from adult subjects (≥ 18 years old) or assent from legal guardians for subjects < 18 years old. The study excluded patients who had been treated for malaria in the 2 weeks preceding a visit to the clinic. Migrant patients were also excluded from participating in the study.

### Sample Collection and Preparation

2–3 ml of blood was collected from eligible candidates who had tested positive by rapid diagnostic test (RDT; Parascreen® (Pan/Pf), Zephyr Biomedicals, Verna Goa, India) for *P. falciparum* malaria. Additionally, FTA filter paper (Whatman Inc., Bound Brook, New Jersey, USA) was used to collect three blood spots of about 100 µl each for DNA extraction. Also prepared were two blood films on glass slides for microscopy. All positive specimens were confirmed by microscopy in the USAMRU-K laboratory.

For immediate *ex vivo* testing, *P. falciparum* isolates from Kisumu district hospital were collected in acid citrate dextrose (ACD) vacutainer tubes (Becton-Dickinson, Inc., Franklin Lakes, New Jersey, USA) and availed to the laboratory within 4–6 hours. *P. falciparum* isolates from Kericho and Kisii district hospitals, were placed in storage-transport media, and refrigerated at 4°C until transported to the laboratory, within 72 hours, for laboratory culture-adaptation.

### *In vitro* Drug Sensitivity Testing

The SYBR Green I-based IC_50_ drug sensitivity assay, described elsewhere [33] was used for *ex vivo* and *in vitro* drug sensitivity testing. Briefly, each isolate was tested against a number of conventional antimalarials namely mefloquine hydrochloride (MQ), Lumefantrine (LU) chloroquine diphosphate (CQ), and amodiaquine hydrochloride (AQ). Drugs were sourced from Walter Reed Army Institute of Research, (Silver Spring, Maryland, USA).

Reference *P. falciparum* clones, D6 (considered CQ-sensitive) and W2 (considered CQ-resistant) were assayed against all drugs as an internal control. These clones were obtained from frozen stocks and culture-adapted for drug sensitivity assays. Stock drug solutions at 1 mg/ml were prepared in 70% ethanol for CQ, MQ and LU or 100% dimethyl sulfoxide (DMSO) for AQ. For starting concentrations complete RPMI 1640 media was used as the diluent, followed by 10 well serial 2-fold dilutions. The following highest and lowest nanomolar (nM) concentration ranges were achieved: AQ (281 to 0.6), CQ (3125 to 6.1), LU (188.7 to 0.37) and MQ (603 to 1.2). The drugs thus prepared were either used immediately, or stored at −80°C for no more than one month.

*P. falciparum* field isolates from Kericho and Kisii, refrigerated in transport media, as well as the 2 *P. falciparum* laboratory reference clones D6 and W2, were culture-adapted before subjecting to the SYBR Green I assay. The parasites were cultured at 6% hematocrit for 7 to 30 days, to reach 3–8% parasitemia [34]. For IC_50_ drug assays, culture-adapted parasites were adjusted to 2% hematocrit and 1% parasitemia, in 96 well plates and antimalarial drug aliquots in complete RPMI 1640 added to the wells.

### *Ex vivo* Drug Sensitivity Testing

Pf isolates from the Kisumu District Hospital, 15 minutes journey from the central lab, were analyzed ex vivo within 4–6 hours of collection. These were processed the day of phlebotomy without culture-adaptation, using the SYBR Green I-based IC_50_ drug sensitivity assay as described in literature [33]. Briefly, blood samples with >1% parasitemia were adjusted to 1% parasitemia at 2% hematocrit, and those with ≤1% parasitemia were used unadjusted at 2% hematocrit. Following this, antimalarial drug aliquots in complete RPMI 1640 were added to the wells and tested as explained above. It was not possible to perform IEV on samples from Kericho District Hospital and Kisii District Hospital as it was not logistically possible to receive these samples within the 4–6 hour window.

### *Pfmdr1* and *Pfcrt* Single Nucleotide Polymorphism (SNP) Analysis and Sequencing for *Pfcrt* 72-76 Haplotype Analysis

SNP analysis was conducted for *Pfmdr1* codons 86 (N86Y) using real-time PCR as previously described [35]. Probes were labeled with the VIC-reporter dye (ABI) for wild type and the FAM-reporter dye for the mutant, respectively. For *Pfcrt,* conventional PCR was done as described elsewhere [27]. Additionally, all PCR amplicons were selected for sequencing. The isolates were purified using QIAquick PCR purification kit (Qiagen Inc). *Pfcrt* sequencing of the amplicons was done on the 3500 xL ABI Genetic analyzer using version 3.1 of the big dye terminator method (Applied Biosystems). Assembling of the generated sequences to make contigs was performed using DNA Baser version 3x and the sequences aligned in MUSCLE version 3.8. The alignment was visualized using BioEdit version 7.1.3.0. All sequences were compared against the 3D7 sequence at the NCBI database.

### *Pfmdr1* Copy Number

For all genotyping assays, DNA was extracted from FTA filter paper blots or whole blood (for *ex vivo* specimens) according to manufacturer instructions (QIAamp DNA Blood Mini Kit, QIAGEN, Inc, Alameda, California, USA). *Pfmdr1* Copy numbers were estimated as previously described [35]. Briefly, genomic DNA from *P. falciparum* reference clone 3D7, known to have 1 copy of *Pfmdr1* gene, was used as the calibrator [36]. The house keeping gene used was *P.falciparum* tubulin, and for multiple *Pfmdr1* copy control, DNA from the Dd2 clone was used.

### Statistical Analysis

We analyzed data by using non-parametric Kruskal-Wallis H one-way ANOVA, the Mann-Whitney U test, Dunn’s Multiple Comparison Test, Chi-square, and Pearson product-moment correlation coefficient (GraphPad Prism 5.00 for windows, GraphPad software, San Diego, CA).

## Results

### Chemosensitivity Assay

A total of 158 West-Kenyan field isolates from Kisumu, Kisii and Kericho were individually assayed for drug sensitivity against CQ, AQ, MQ and LU between 2008 and 2011. The data were then pooled and analyzed. The four drugs were also tested against D6 and W2, which serve as CQ sensitive and CQ resistant reference strains, respectively. For D6 the median IC_50_ values in nM units were as follows: CQ, 13.0, *n = 14,* (Interquartile range (IQR) 5.3 to 18.3), MQ; 86.9, n* = 11* (IQR 61.2 to 125.1), LU; 8.0, *n = 12* (IQR 6.0 to 12.5), AQ; 3.3, n* = 9* (IQR 2.8 to 4.3). Additionally, we report the following median IC_50s_ in nM against W2: CQ; 209.8, *n = 9*, (IQR 194.7 to 273), MQ; 5.5 n = 12 (IQR 4.1 to 7.8), LU; 45.1 *n* = 5 (IQR 28.8 to 92) and AQ; 21.0, *n* = 7, (IQR 15.6 to 30.2).

Median IC_50_ values for the field isolates for the four drugs were considered by year (Table 1). AQ median IC_50_ values decreased significantly between 2008; 14.5 nM, *n = 51* (IQR 6.7 nM to 21.5 nM) and 2011; 5.7 nM, *n = 61* (IQR 2.9 nM to 8.7 nM) (p<0.0001). We also observed a significantly steady decline of median CQ IC_50_ from a high of 92.8 nM, *n = 49* (IQR 39.5 nM to 163.3 nM) in 2008 to a low of 22.4 nM, *n = 53* (IQR; 13.0 nM to 92.4 nM) in 2011 (p<0.0001) (Table 1). MQ showed a median of 17.4 nM, *n = 45* in 2009 (IQR; 10.2 nM to 38.3 nM) and a median of 24.7 nM, *n = 61* (IQR; 10.6 nM to 39.5 nM) in 2011. Comparatively, LU showed a median IC_50_ of 23.9 nM, *n = 51* in 2009 (IQR; 15.3 nM to 45.9 nM) and a median of 31. nM, *n = 52* (IQR; 9.5 nM to 52.4 nM) in 2011. However, the changes for MQ and LU for the study period did not attain statistical significance (p values of 0.07 and 0.17, respectively).

**Table 1:** Median IC_50s_ for CQ, AQ, LU and MQ per Year during 2008–2011.

Year	CQ (n)	AQ (n)	LU (n)	MQ (n)
2008	92.81(49)	14. 49(51)	22.91(11)	18.11(45)
2009	69.30(39)	7.98(42)	23.88(51)	17.42(45)
2010	44.91(51)	5.11(41)	32.08 (50)	19.52(48)
2011	22.37(53)	5.66(61)	31.56(52)	24.7(61)
p value	*p<0.0001*	*p<0.0001*	*P = 0.17*	*p = 0.07*

Medians were calculated in Graphpad prism Version 5. Statistical significance was determined using Kruskal Wallis H test.

### IC_50_ Comparison against *Pfcrt* K76T and *Pfmdr1* N86Y Genotypes

**Figure 1:**
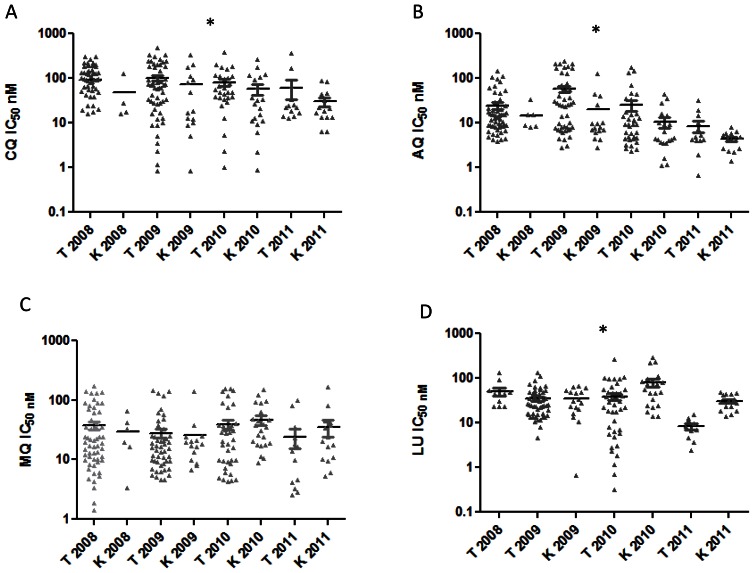
*Pfcrt* K76T SNP compared against drug IC_50_ (in nM). A. Comparison of *Pfcrt* K76T SNP against CQ IC_50_ stratified by year; B. Comparison of *Pfcrt* K76T SNP against AQ IC_50_ stratified by year; C. Comparison of *Pfcrt* K76T SNP against MQ IC_50_ stratified by year; D. Comparison of *Pfcrt* K76T SNP against LU IC_50_ stratified by year. Median values are shown. *indicates data is significant.

CQ IC_50s_ were compared against *Pfcrt* K76T and *Pfmdr1* N86Y between 2008 and 2011 for all samples that had successfully been analyzed for both SNPs and IC_50_ values (Kruskal-Wallis H test and Dunn’s multiple comparison test). When considered inter-year, PfCRT-K76 carrying parasites (labeled by “K” and the year) were significantly sensitive to CQ as compared to those with 76T (labeled by “T” and the year) (Figure 1A) as follows: T 2008 vs K 2010; p<0.01 and T 2008 vs. K 2011; p<0.001. Additionally, in 2011 parasites with 76 T were more sensitive to CQ when compared to 76T carrying parasites in 2008 (p<0.05 Figure, 1 B). On the contrary, no significant relationships were established between *Pfmdr1* N86Y and CQ IC_50_s during the same period.

**Figure 2:**
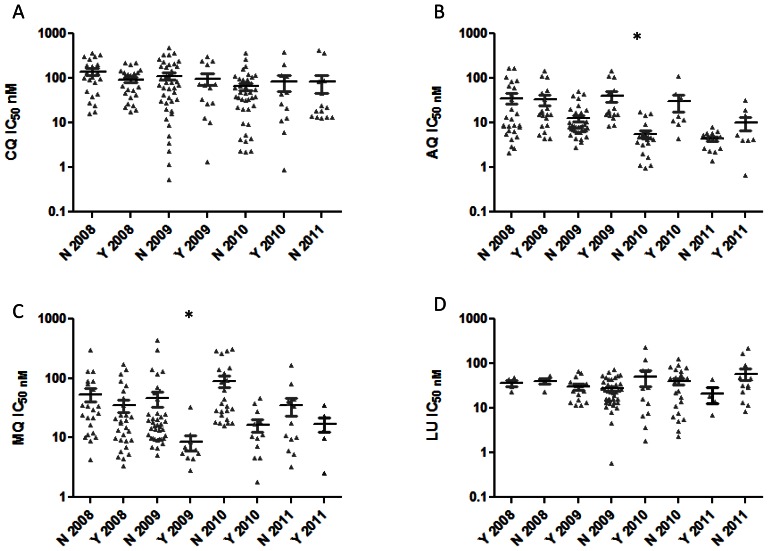
*Pfmdr1* N86Y SNP compared against drug IC_50_s (in nM). A. Comparison of *Pfmdr1* N86Y SNP against CQ IC_50_ stratified by year; B. Comparison of *Pfmdr1* N86Y SNP against AQ IC_50_ stratified by year; C. Comparison of *Pfmdr1* N86Y SNP against MQ IC_50_ stratified by year; D. Comparison of *Pfmdr1* N86Y SNP against LU IC_50_ stratified by year. Median values are shown. *indicates data is significant.

When AQ median IC_50s_ were analyzed based on *Pfcrt* genotypes, K76 related medians were significantly different from those of 76T between years (Figure 1B). Thus, we observed inverse inter-year relationships as follows: T2008 vs. K2010; p<0.05, T2008 vs K2011; p<0.001, T2009 vs. K2010; p<0.001, T2009 vs. K2011; p<0.001 and T2010 vs. K2011; p<0.05. Moreover, the AQ 76T related median IC_50_ was significantly different between 2009 and 2011, p<0.001 (Figure 1B). When a similar analysis was done for AQ and *Pfmdr1* N86Y, we found that AQ median IC_50_ values were also significantly associated with N86Y both intra- and inter-year (Figure 2B). Thus, we observed the following significant inter-year inverse relationships between 86N and 86Y related median AQ IC_50_s: Y2008 vs. N2010, p<0.001; Y2008 vs. N2011, p<0.001; Y2009 vs. N2010, p<0.001; and Y2009 vs. N2011 p<0.001.

In regard to MQ, we observed the following significant inter−/intra-year inverse associations between N86 and 86Y related medians (Figure 2C): N2008 vs. Y 2009, p<0.01; Y2008 vs. N2010, p<0.05; Y2009 vs. N2010, p<0.001; Y 2009 vs. N 2009, p<0.05 and Y2010 vs. N2010, p<0.01. On the contrary, there was no discernible relationship between MQ and *Pfcrt* K76T. This study also showed significant intra−/inter-year relationships between LU and K76T, whereby K76 isolates had comparatively higher LU median IC_50_ than 76T isolates (Figure 1D) as follows: K2009 vs. T2011, p<0.01; T2010 vs. K2010, p<0.05; K2010 vs. T2011, p<0.001; T2011 vs. K2011, p<0.05. Additionally over the study period 76T related medians were different as follows: T2008 vs. T2011, p<0.001; T2009 vs. T2011, p<0.01; and T2010 vs. T2011, p<0.001. It was observed that the LU 76T median IC_50s_ of 2011 were much lower than the preceding years (Figure 1D). Interestingly, we did not observe any relationships between LU median IC_50_ values and *Pfmdr1* N86Y during the study period. Equally, *Pfmdr1* gene amplification was not discerned in any of the study samples.

### *Pfcrt* 72-76 Haplotypes during 2008–2011

**Figure 3:**
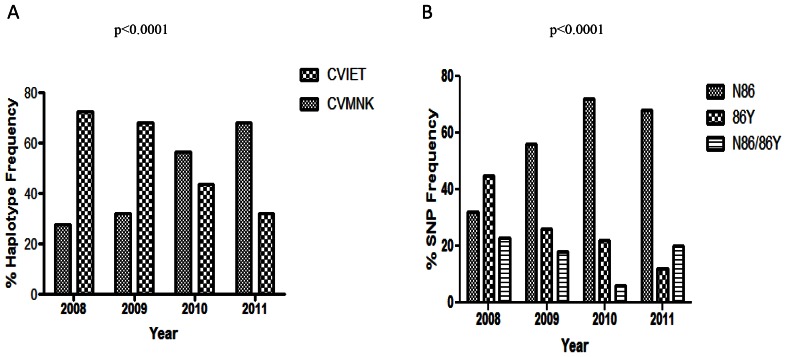
Frequency of *Pfcrt* 72-76 haplotype and *Pfmdr1* N86Y SNP. A. *Pfcrt* 72-76 haplotype frequency per year; B. *Pfmdr1* N86Y SNP frequency per year. Statistical significance was determined by Chi-square.

We investigated the *Pfcrt* 72-76 haplotypes in all samples that were successfully sequenced between 2008 and 2011 (n = 333). In 2008 we assayed 87 samples, of these, 27.59% were CVMNK and 72.4% were CVIET at the 72-76 respective positions. During 2009 a total of 69 samples were analyzed of which 31.9% had the CVMNK haplotype whereas 68.1% were CVIET. In 2010 out of 124 isolates 56.5% were CVMNK and those with CVIET were at 43.6%. In 2011, a total of 53 samples were assayed with 67.9% carrying the CVMNK haplotype compared to CVIET at 32.1%. We did not observe the SVMNT haplotype that is associated with high levels of AQ resistance among the study samples. There was a significant percentage change of the CQ mutant haplotype CVIET, to the CQ sensitive CVMNK haplotype between 2008 and 2011 (p<0.0001; Figure 3A) during the study period.

### *Pfmdr1* N86Y Frequencies between 2008 and 2011

We analyzed frequencies of SNPs in *Pfmdr1* codon 86 in 243 samples during 2008, 225 samples in 2009, 395 samples in 2010 and 314 samples in 2011. We observed a steady increase in the frequencies of *Pfmdr1* N86 genotype as compared to 86Y and N86/86Y between 2008 and 2011 (p<0.0001, Figure 3B).

### In vitro Drug Activity Correlation Tests

Using the Pearson product-moment correlation test we sought to further delineate any relationships among the test drugs (Table 2). We found no correlation between the two 4-amino Quinolines, AQ and CQ, phenotypic activity (r = 0.009, p = 0.92). However, a positive correlation between phenotypic activities of the two aryl amino-quinoline alcohols LU and MQ (r = 0.516, p<0.0001) was detected. There was a moderate inverse correlation between LU and CQ (r = −0.3, p = 0.002).

**Table 2:** Pearson product-moment correlation for CQ, AQ, MQ and LU.

Drug pair	r	r^2^	p- value
AQ-MQ	0.1999	0.03996	0.0266
AQ-LU	0.2058	0.04234	0.0267
AQ-CQ	0.0091	0	0.9199
LU-MQ	0.516	0.2663	<0.0001
LU-CQ	−0.3004	0.09026	0.0028
MQ-CQ	0.11	0.01209	0.2318

Correlation was determined in Graphpad prism Version 5 based on IC_50_ values for each pair of drugs per isolate.

### Analysis of Combined Pfcrt 76/Pfmdr1 86 Haplotypes vs. Medians

We analyzed Pfcrt 76/Pfmdr1 86 haplotypes in the following combinations: K76-N86, K76-86Y, 76T-N86, 76T-86Y. These were compared against the respective AQ, CQ, LU and MQ Median IC50 (Table 3). For CQ, we observed that samples carrying the haplotypes K76-N86 and K76-86Y had the lowest medians at 18.1 nM and 18.8 nM, respectively, as compared to 76T-N86 and 76T-86Y at 70.3 nM and 71.3 nM, respectively (p<0.0001, Table 3). Samples with the haplotype K76-N86 were the most sensitive to AQ as compared to the other three haplotypes (p<0.01, Table 3). For LU, there were no significant differences among samples in the four haplotypes categories when compared against their respective median IC50 values. For MQ, the K76-86Y and 76T-86Y haplotypes carrying samples showed significant differences in their median IC50 values when compared against 76T-N86 and K76-N86 (p<0.001, Table 3).

**Table 3:** Combined Pfcrt 76/Pfmdr1 86 haplotype vs. medians.

Medians in nM (n)					
	K76-N86	K76-86Y	76T-N86	76T-86Y	P Value
CQ	18.8 (62)	18.1 (21)	70.3 (56)	71.3 (34)	<0.0001
AQ	5.9 (35)	11.2 (15)	8.4 (34)	8.4 (30)	<0.01
LU	33.5 (70)	33.1 (17)	27.2 (56)	22.8 (30)	0.5
MQ	22.2 (82)	15.2 (23)	21 (70)	9.2 (37)	<0.001

Medians were calculated in Graphpad prism Version 5. Statistical significance was determined using Kruskal Wallis H test. Comparisons were done using Dunns Multiple comparison test.

## Discussion

We have characterized *P. falciparum* field isolates from Western-Kenya during the period 2008–2011 based on analysis of SNPs in *Pfmdr1* and haplotypes in PfCRT. This analysis was done in relation to susceptibility profiles of four antimalarial drugs namely CQ, AQ, MQ and LU, for the same period. *Pfcrt* 76T has previously been implicated in chloroquine resistance as well as LU tolerance [32]. This study has shown rapid conversion of the parasite population to the CQ sensitive allele K76 between 2008 and 2011. This time period coincides with the comprehensive use of Coartem in Kenya for malaria treatment, which was partly driven by the ease of availability starting in 2008 [37]. We suggest that reciprocate reduction in the use of AQ caused a release of drug resistance pressure on *Pfcrt* (Figure 1B) and *Pfmdr1* (Figure 2B). This trend is supported by the observed rapid increase in both AQ and CQ sensitivity (Table 1). Prior to 2008, CQ resistance was observed for a long time even after stoppage of its use in Kenya in 1998. In fact, a study looking at the *Pfcrt* changes over a 13 year period between 1993 and 2006 in Kilifi, Kenya found that the 76T mutation only decreased from 94% to 63% [38]. A study in Malawi described a larger reduction–from 85% to 13%–over a similar time period, which may suggest that CQ related drug resistance pressure may have been continued at higher rates in Kenya as compared to elsewhere in Africa. Our study measures a more recent timeframe and shows a reduction from 77% to 30% over a period of 4 years (2008–2011) accompanied by increased CQ and AQ phenotypic drug sensitivity. We speculate that the significant widespread use of AQ in Kenya as an alternative to S/P prior to the use of ACTs may have resulted in the observed apparent maintenance of CQ resistance due to structural similarities between the two drugs [39], but with the advent and acceptability of ACT use within the Kenyan health care community this drug pressure dissipated in the last 5 years.

Haplotypes in *Pfcrt* at the 72–76 loci have been linked to both AQ and CQ resistance. Specifically, the SVMNT variant has been linked to high AQ resistance and moderate CQ resistance [31]. As expected, the highly CQ resistant haplotype CVIET which has been linked to resistance in Southeast Asia and Africa, was the most observed during the year 2008 while CVMNK linked to CQ sensitivity was the least observed in 2008. However there was a dramatic and steady reversal of the relative status of the two haplotypes between the year 2008 and 2011 (Figure 3A). No correlation between AQ and CQ was observed as measured by the Pearson product-moment correlation test (Table 2). We did not detect the SVMNT haplotype in our study samples, but it has previously been reported in Tanzania [40] and most recently in Angola [41]. In the case of Tanzania, the acquisition of the SVMNT resistance haplotype was rapid (between 2003 and 2004), and it is unclear why we did not observe this haplotype in western Kenya. However, pre-2008 isolates will be tested to establish its presence/absence prior to this study period. The absence of SVMNT–an indicator of AQ sensitivity–is potentially fortuitous since the amodiaquine-artesunate (ASAQ) combination is a potential ACT alternative to Coartem in Kenya. Indeed studies have proven that ASAQ has satisfactory efficacy against *P.falciparum* in Kenya [42], [43].

The present data shows that parasites carrying the wild type *Pfcrt* allele, K76, had a significantly higher median LU IC_50_ value compared to isolates with 76T beginning in 2009 (Figure 1D). It has previously been suggested that increased sensitivity to chloroquine would be accompanied by resistance to LU [17]. In Kenya, LU has been shown to select for the K76 allele [17]. Based on changing alleles in Pfcrt we suggest that reestablishing of LU IC50 baselines may be occurring.. Analysis of IC_50_ values show that there is significant positive correlation between LU and MQ (Table 2), an indicator of cross resistance between the two drugs. In addition, previous work has shown increasing MQ tolerance in Kenya in the absence of drug pressure [33], [44]. Studies in East Africa have demonstrated MQ selection of the *Pfmdr1* N86 resistance gene [32], [45]. This study shows that between 2008 and 2011, there has been a substantial rise in the prevalence of N86 allele (Figure 3 B) among our specimens. Even though there was an increase in MQ median IC_50_ values between 2008 and 2011, this increase did not attain statistical significance. Copy number amplification of the *Pfmdr1* gene has been shown to cause MQ resistance [22]. We did not observe multiple copy numbers of *Pfmdr1* in any of our isolates, but we will continue to monitor *Pfmdr1* copy numbers in western Kenya in correlation with sensitivity and SNP data.

*Pfmdr1* SNP changes in Kenya have been previously studied for the periods 1999–2000 and 2003–2005 [1], [44]. Comparing these two periods, an increase in the prevalence of the 86Y mutation is observed. Data for the year 2008 [33] show that the prevalence rates of N86Y SNP remained unchanged as compared to those of 2003–2005 [44]. However, in the present, study covering the period 2008 to 2011, we observe a steady decrease in codon 86 mutation rates (Figure 3B). Drug policy change in Kenya from S/P to AL (Coartem) was announced in 2006 and broadly implemented by 2008, countrywide with support from the Global Fund [37]. Thus, it would be expected that co-resistance between MQ and LU may explain the observed trends of N86Y in our study beginning in the year 2009, stemming from LU drug pressure. However, it is noteworthy that whereas there are trends showing significant association between MQ and N86Y over the study period, none can be established between LU and N86Y (Figure 2D). Thus, we speculate that there are other factors that may be involved with the changes observed in *Pfmdr1* N86Y.

Interestingly, beginning 2009 there was a significant trend linking AQ to changes in *Pfmdr1* N86Y. AQ has been marketed in Kenya as an over-the-counter medication following widespread resistance to S/P and prior to widespread ACT use [2]. It would appear that availability of the cheaper AL (Coartem), driven by support from the Global Fund beginning 2008, significantly decreased the demand and usage of the more expensive AQ. This conjecture is supported by the observation of a rapid increase in AQ susceptibility between 2008 and 2011 (Figure 1B, Table 1). Therefore, whereas LU may modulate the parasite to institute resistance against MQ due to the physicochemical similarities in the two drugs, AQ may do the same through changes in *Pfmdr1*. This issue is more than academic as both AQ and LU have been combined with ART and are currently marketed in Kenya for malaria chemotherapy. The continued use of the two drugs may indirectly contribute towards mefloquine tolerance in Kenya. Finally, as expected a combination of four Pfcrt 76 and Pfmdr1 86 haplotypes namely K76-N86, K76-86Y, 76T-N86, 76T-86Y in comparison to CQ median IC50 value show that, K76T is the most important allele in CQ drug response (Table 3). This study has confirmed that the N86Y allele is critical in MQ drug response (Table 3).

### Conclusion

Our data indicate that the changing drug policy during 2008–2011, which provided the ACT, in the form of CoArtem, on subsidy in private retail shops and freely in public hospitals, had an effect on drugs used during the same period. Subsequently, AQ and CQ showed increasing sensitivity from 2008 to 2011. Concordantly, *Pfcrt* and *Pfmdr1* the resistance markers for CQ, AQ and MQ showed a rapid conversion to wild types. Since MQ and LU show positive correlation, co-resistance between the two drugs is largely expected, and therefore increased use of AL may precipitate tolerance to both drugs. Results in this study implicate AQ in modulating parasite resistance towards CQ, LU and MQ via changes in *Pfcrt* and *Pfmdr1*. Continued surveillance is therefore required to monitor resistance profiles of the four drugs.
